# ST elevation myocardial infarction during head-up tilt testing

**DOI:** 10.34172/jcvtr.2020.12

**Published:** 2019-10-05

**Authors:** Lucas Simonetto Faganello, Mauricio Pimentel, Ana Paula Arbo Magalhães, Leandro Ioschpe Zimerman

**Affiliations:** ^1^Post-Graduate Program in Cardiology and Cardiovascular Sciences, Medical School, Universidade Federal do Rio Grande do Sul, Porto Alegre, Brazil; ^2^Cardiac Electrophysiology Group, Cardiology Division, Hospital de Clínicas de Porto Alegre, Porto Alegre, Brazil

**Keywords:** Syncope, Head-up Tilt Testing (HUTT), ST Elevation Myocardial Infarction

## Abstract

We report a case of ST elevation myocardial infarction (STEMI) during head-up tilt testing (HUTT). A 54-year-old man was admitted to our emergency department after four episodes of syncope. Treadmill test and electrophysiological study were normal. During passive HUTT, the patient had inferolateral ST elevation. Coronary angiography showed two severe lesions in the right coronary artery and circumflex artery.

## Introduction

Head-up tilt testing (HUTT) is a useful diagnostic tool for syncope investigation,^1^ and few complications are expected. Arrhythmias, ST-segment elevation due to coronary vasospasm, non-ST elevation myocardial infarction (non-STEMI),^2^ and recently, ventricular fibrillation^3^ were reported after drug sensitization. As far as we know, this is the first report of ST elevation myocardial infarction (STEMI) during HUTT.

## CaseReport

**Figure 1:**
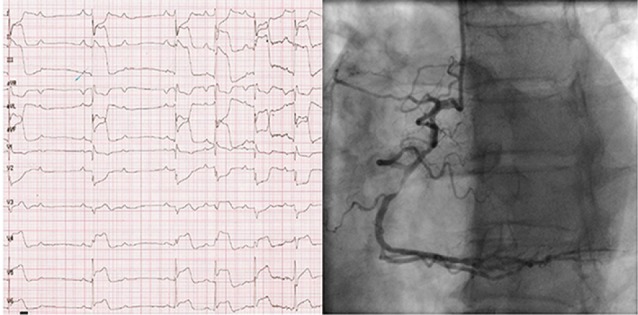
12-lead electrocardiogram recorded during the HUTT showing a marked inferior and lateral ST-elevation with a reciprocal ST depression in the anterior leads and a 2º degree AV block. Coronary angiography showing a severe lesion of 95% in the middle segment of right coronary artery.

A 54-year-old man was admitted to our emergency department after a syncopal episode, the fourth in a week. All of them were preceded by lightheadedness, blurred vision and sweating, and one of them was also preceded by chest pain. The first episode occurred while he was hanging clothes on a clothesline, and he recovered after two minutes. The following episodes occurred while he was walking. Despite the reported smoking load of 20 packs/year and history of hypertension, he had no documented cardiac disease nor family history of syncope or sudden death. Physical examination showed normal vital signs, no orthostatic hypotension and grade III systolic ejection murmur at the left lower sternal border without radiation. Electrocardiogram (ECG) showed sinus rhythm, normal axis, PR interval of 150 ms, QRS duration of 112 ms, incomplete right bundle branch block and no ST-segment displacement. Echocardiography revealed moderate tricuspid valve regurgitation due to a primary disease, and normal left ventricle. Considering that syncope episodes occurred during walking a treadmill test was ordered. Treadmill test was normal (11.7 METs). Electrophysiological study showed normal sinus and atrioventricular (AV) function and no tachyarrhythmia was induced. HUTT was performed, and after 8 minutes of passive tilt, blood pressure (BP) started dropping. The patient showed dizziness, nausea and profuse sweating. After 12 minutes, BP was 70/40 mm Hg, and ECG showed marked and progressive ST elevation in leads II, III, AVF, V4, V5 and V6 with reciprocal ST-segment depression in V1 and V2. The patient lost consciousness and was tilted back to supine position. Immediately, he regained consciousness and referred intense chest pain, while ST elevation got worse and 2:1 AV block appeared (Figure 1A). After 5 minutes, the patient was asymptomatic, with normal ECG. Urgent coronary angiography showed atherosclerotic plaques resulting in 95% obstruction in the middle segment of right coronary artery (Figure 1B) and 80% obstruction in the middle segment of circumflex artery. Coronary angioplasty was performed, and drug-eluting stents were successfully implanted. Initial high-sensitive cardiac troponin T (hs-cTnT) levels obtained after 1 hour of procedure was 53,42 ng/L (normal range <14 ng/L). Subsequent hs-cTnT obtained after 12 hours of procedure was 66,11 ng/L. No syncopal episodes were reported on 6-month follow-up.

## Discussion

Neurally-mediated syncope is the most common type of syncope.^4^ Although its triggers and clinical presentation are well known, sometimes this condition may be associated with other disorders, such as myocardial ischemia, leading to an atypical presentation.^5,6^ We reported a case of STEMI during HUTT in a patient with normal treadmill test results two days earlier. The patient had no history of syncope until one week before hospitalization, and one episode was preceded by chest pain. We believe that hypotension due to vasodepressor reflex unmasked the coronary disease, leading to typical STEMI. The sensitivity of treadmill test for diagnosing coronary heart disease is 45%-61% which could explain a previously normal test.^7^ In this case we consider that vasodepressor reflex was an unusual trigger for acute myocardial ischemia. To the best of our knowledge, occurrence of STEMI during passive HUTT has not been reported previously in the literature.

## Competing interests

None declared.

## Ethical approval

Informed consent has been obtained from the patient to publish this material.
